# Recruiting to Clinical Trials on the Telephone – a randomized controlled trial

**DOI:** 10.1186/s13063-016-1680-y

**Published:** 2016-11-21

**Authors:** Kim Thestrup Foss, Jesper Kjærgaard, Lone Graff Stensballe, Gorm Greisen

**Affiliations:** 1 Department of Paediatrics and Adolescent Medicine, Research Unit Women’s and Children’s Health, Juliane Marie Centre, Copenhagen University Hospital Rigshospitalet, Blegdamsvej 9, 2100 Copenhagen Ø, Denmark; 2 Neonatal Department, Juliane Marie Centre, Copenhagen University Hospital Rigshospitalet, Blegdamsvej 9, 2100 Copenhagen Ø, Denmark

**Keywords:** Recruitment, Comprehension, Satisfaction, Informed consent, Telephone, Randomized controlled trial, Clinical trial

## Abstract

**Background:**

Informed consent is an essential element of clinical research. Obtaining consent, however, may be challenging. The use of the telephone for giving information and obtaining consent may be practical but little formal research has been done.

**Methods:**

We examined the use of the telephone for the purpose of informing expectant mothers about The Danish Calmette Study; a randomized clinical trial assessing neonatal Bacille Calmette-Guérin vaccination. Expectant mothers received an invitation letter with a Participant Information Sheet about The Danish Calmette Study, the present trial, and a Consent Form. Two to 4 weeks later we contacted the mothers to discuss potential participation in the present trial. At this initial telephone contact, and after consent from the mothers, we randomized expectant mothers to receive the verbal information about The Danish Calmette Study by telephone, or at a face-to-face consultation. The primary outcome was a communication score, consisting of comprehension of information about The Danish Calmette Study and satisfaction with the information process. The outcome was measured using a questionnaire 2 days after the information was provided, and 2.5 months after the birth of the child.

**Results:**

The communication score obtained 2 days after information was given was significantly reduced in the telephone group, effect size −0.74 (95% confidence interval (CI), −1.11 to −0.36). The effect sizes of the subscores were −0.87 (95% CI, −1.25 to −0.49) for satisfaction and −0.22 (95% CI, −0.58 to 0.14) for comprehension. The effect sizes were slightly reduced when assessed 2.5 months after the birth.

**Conclusion:**

The communication score was reduced in the telephone group. This was due to a reduction in satisfaction, while no difference in the comprehension could be found in comparison to the control group. This may be ethically acceptable as both groups had high satisfaction scores.

**Trial registration:**

ClinicalTrials.gov, registered on 5 October 2015 with trial registration number NCT02570061.

**Electronic supplementary material:**

The online version of this article (doi:10.1186/s13063-016-1680-y) contains supplementary material, which is available to authorized users.

## Background

Informed consent is a requirement for clinical research. The process leading to informed consent should provide potential participants – or their guardians – with sufficient information to make informed decisions about their voluntary participation in research projects [1]. This is one of the major principles stated in the Declaration of Helsinki, which is the basis of rules and regulations concerning informed consent [1–3].

Randomized clinical trials are considered the “gold standard” for evaluating the effect of health care interventions. In spite of this, many of them do not meet their recruitment targets [4]. Slow recruitment can lead to prolonged study duration, increased resource use, or premature termination of recruitment that can result in underpowered studies [4, 5]. Therefore, improvement of the informed consent process should always be pursued [4, 6–8]. Informing potential participants or guardians and obtaining informed consent by telephone could be more effective than informing face-to-face, which is the usual strategy preferred by the Ethical Review Boards [9].

We wanted to examine the effect of providing study information on the telephone compared to a face-to-face consultation in a randomized controlled study. The outcome measures were study comprehension and satisfaction with the information process. We utilized the recruitment process for a large randomized trial – The Danish Calmette Study – evaluating the nonspecific effects of Bacille Calmette-Guérin vaccination at birth [10].

## Methods

### Trial design and participants

We enrolled expectant mothers who were also potential participants in The Danish Calmette Study in a parallel-group randomized controlled design. Inclusion criteria were eligibility for The Danish Calmette Study, which is described in details elsewhere [10], with the exceptions that gestational age and birth weight of the child were unknown, and the Consent Form for The Danish Calmette Study was not yet signed at the time of the inclusion in the present trial. Exclusion criteria were contact with the study staff prior to enrollment in the present trial or not being able to meet in person for a consultation. Enrollment took place from July to November 2013. Follow-up took place from July 2013 to February 2014.

### Trial setting within The Danish Calmette Study

The Danish Calmette Study was a multicenter study in Denmark. The process of obtaining informed consent in The Danish Calmette Study had three steps: firstly, all parents planning to give birth at Rigshospitalet, Hvidovre Hospital and Kolding Sygehus received a letter during the 2nd or 3rd trimester with an invitation to enroll their newborn in The Danish Calmette Study. The letter contained information about The Danish Calmette Study, along with general information about participant rights in medical research, a Consent Form, and information regarding the present trial. Secondly, after 2 to 4 weeks, study staff called the family to elaborate on the information, assess eligibility, and discuss the potential participation in The Danish Calmette Study. Parents were given the opportunity to take more time for consideration, or schedule a meeting with the study staff prior to giving consent. And thirdly, if the parents decided to participate in The Danish Calmette Study, they signed the Consent Form and returned it. The Research Ethics Committee gave permission for obtaining informed consent for The Danish Calmette Study by telephone, although the committee clearly recommends face-to-face meetings [9].

**Fig. 1:**
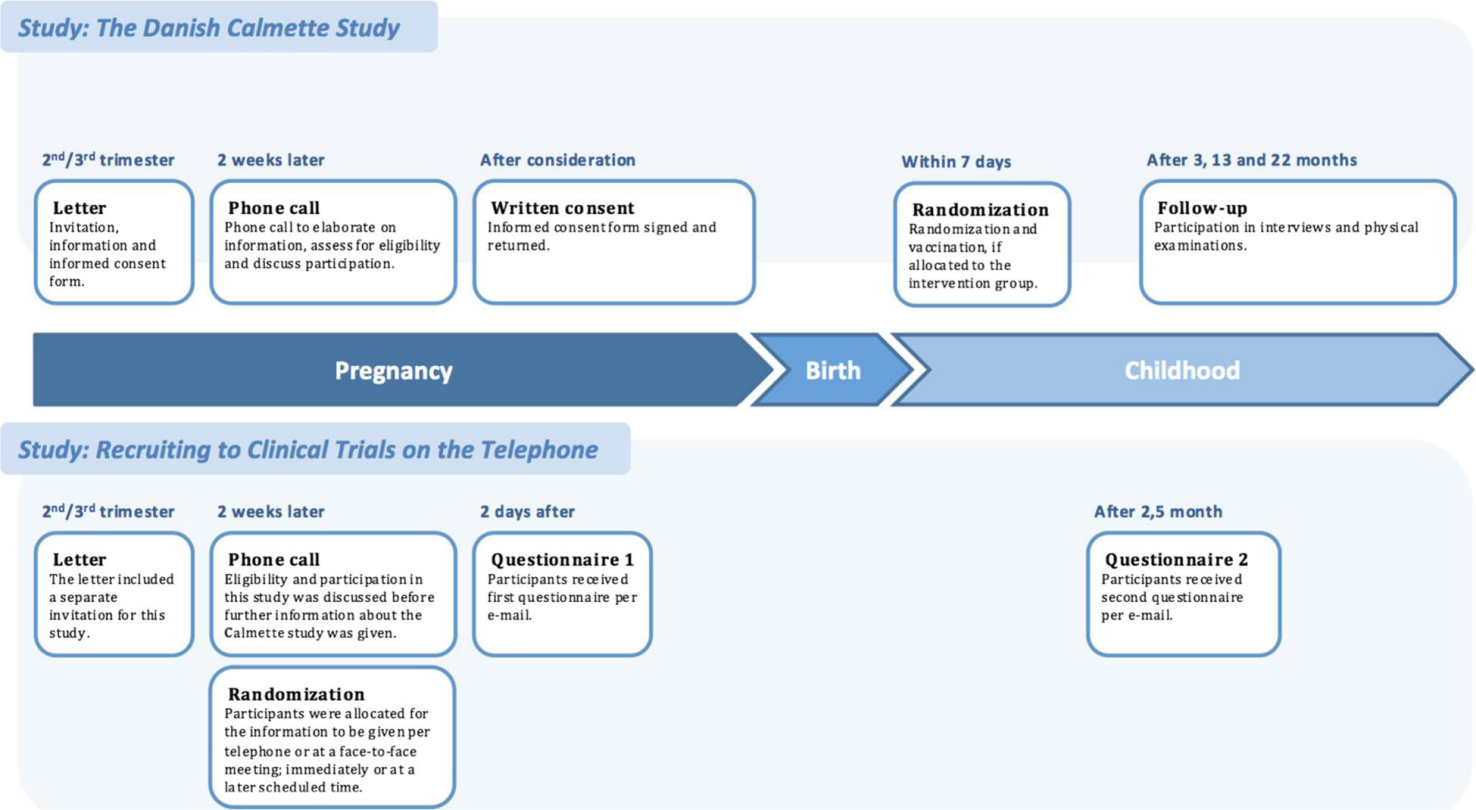
Timeline for The Danish Calmette Study and Recruiting to Clinical Trials on the Telephone

Expectant mothers planning to give birth at Rigshospitalet were also invited for the present trial (Fig. 1). They received a Participant Information Sheet about the present trial along with the one about The Danish Calmette Study. The enrollment for the present trial stopped when the inclusion for The Danish Calmette Study ended.

### Intervention and randomization

At the initial telephone contact we discussed the mothers’ potential participation in the present trial and they gave verbal informed consent before we enrolled them (Fig. 1). After verbal informed consent, we randomized expectant mothers to receive all verbal information about The Danish Calmette Study either on the telephone (telephone group) or at a face-to-face consultation (control group) in a 1:1 ratio. Participants were randomized immediately after verbal informed consent. We used computer-generated block-randomization with block sizes of 2, 4, and 6 to allocate participants to one of the two groups before any further information about The Danish Calmette Study was given. Participants who were randomized to the telephone group continued the conversation on the telephone immediately or at a later scheduled time. Participants randomized to the control group made an appointment to receive the information at a consultation at the hospital and no further information was provided on the telephone.

In the present trial, three study staff provided information on both the telephone and at consultations to approximately the same number of participants in both groups. This was done to minimize the risk of bias due to personal characteristics of the study staff. We used a Standard Operating Procedure to ensure that the same formal information was provided to both groups. The Standard Operating Procedure strictly followed the Committee on Health Research Ethics’ guidelines for verbal information while obtaining informed consent [3]. The time spent providing information and respond to questions was recorded.

### Measurement of comprehension and satisfaction

Participants received an electronic questionnaire by e-mail 2 days after randomization where the information was given (Questionnaire 1) and 2.5 months after the participant gave birth (Questionnaire 2) (Fig. 1). The questionnaires corresponded to the validated Quality of Informed Consent (QuIC) [11] questionnaire, but were reduced in scope and contained items on more specific legal aspects of informed consent and items designed to assess to what degree the guidelines about verbal information from the Committee on Health Research Ethics were followed [3, 12, 13]. Additionally, they contained items on whether a family member or friend was present when the information was provided, and whether the participant had sought additional information regarding The Danish Calmette Study. The questionnaires were divided into six categories; five on study comprehension and one on satisfaction with the information process (see questionnaires in Additional file 1). The items in the first five categories could be answered with “yes,” “no” or “do not know.” The last category was rated on a 7-point Likert scale, with 1 being “very dissatisfied” and 7 being “very satisfied.” Participants filled out the questionnaires online and received the correct answers after finishing the questionnaire in order to correct any misperceptions that they might have had. All questions were required to be answered in order to submit the questionnaire.

### Outcomes

The primary outcome, named communication score, was the sum of the score for comprehension items and satisfaction items from Questionnaire 1. Comprehension items were scored 1 point for each correct answer and 0 points for each incorrect answer. Satisfaction items were scored as rated on the 7-point Likert scale. The total score ranged from 7 to 69 points, the comprehension score from 0 to 20 points, and the satisfaction score from 7 to 49 points.

### Ethics and registration

The Danish Calmette Study was approved by the Committee on Health Research Ethics, the Danish Health and Medicines Authority, the Danish Data Protection Board, and registered at EudraCT and ClinicalTrials.gov (https://clinicaltrials.gov) [14]. The protocol of the present trial was submitted to the Committee on Health Research Ethics in Denmark (file no. H-4-2013 FSP-044) but was classified as a survey and thus not requiring approval. This trial was registered at ClinicalTrials.gov on 5 October 2015 with trial registration number NCT02570061.

### Statistical methods

A sample size of 154 participants (77 in each group) was required to detect an effect on the primary outcome of 0.45 standard deviation (SD) scores with a two-sided 5% significance level and a power of 80%. The analysis strategy was completers’ analysis, so participants lost to follow-up or with missing information in the questionnaires were excluded. We used Spearman’s correlation for scores of comprehension and satisfaction. Cronbach’s *α* was used to judge the appropriateness of combining the scores of comprehension and satisfaction into a single score. The outcome scores were sufficiently normally distributed (i.e., skewness < |2| and kurtosis < |9|) to perform a parametric *t* test. An independent *t* test was used to analyze differences in scores between the telephone and the control group. A Welch’s *t* test was performed when variances were unequal. Effect size was quantified as Cohen’s *d* since the scores themselves are arbitrary. Participants who did not reply to both questionnaires were excluded from analysis.

## Results

**Fig. 2:**
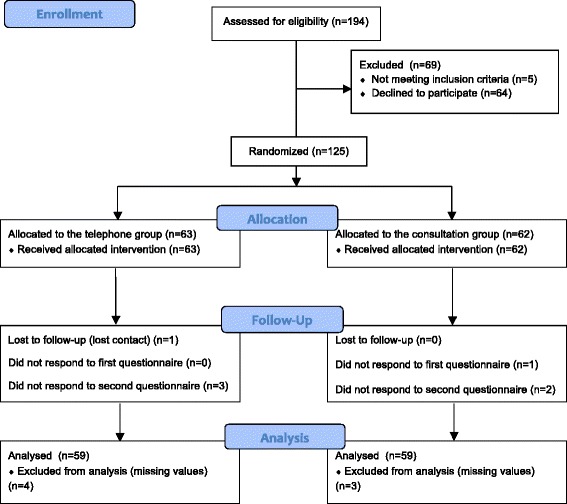
Flow diagram of participants. Participants who have had contact with The Danish Calmette Study staff before being invited for this trial or were unable or declined to meet for a consultation were excluded before randomization. Participants not responding to both questionnaires were excluded in the analysis

Of the 473 expectant mothers invited for the present trial, 279 (59.0%) were not assessed for eligibility because they did not want to participate in The Danish Calmette Study, or because contact could not be achieved. Of the remaining 194 assessed for eligibility, 69 (35.6%) were excluded: five did not meet the inclusion criteria and 64 declined to participate. We randomized 125 expectant mothers. Overall, adherence was high 118 (94.4%). However, seven participants were lost to follow-up; one could not be reached within the follow-up period and six participants replied to only one of the two questionnaires (Fig. 2). In our data with 118 participants (59 + 59) we were able to detect a difference in means of 0.52 and a SD of the difference of 1 with a two-sided 5% significance level and a power of 80%. Table 1 shows the baseline characteristics of the population in the present trial.

**Table 1:** Baseline participant characteristics

	Telephone group (*n* = 59)	Control group (*n* = 59)
Age – median (IQR), years	31 (29 to 34)	32 (28 to 35)
Education – number (%)		
Basic schooling and nontheoretical education	6 (10.2)	8 (13.6)
Theoretical education including bachelor’s degree or equivalent	20 (33.9)	20 (33.9)
Master’s degree or equivalent or more	33 (55.9)	31 (52.5)
Social status – number (%)		
Living with partner	55 (93.2)	55 (93.2)
Not living with partner	4 (6.8)	3 (5.1)
Unknown	0 (0)	1 (1.7)
Prior knowledge of The Danish Calmette Study from antenatal classes – number (%)		
Yes	19 (32.2)	16 (27.1)
No	39 (66.1)	41 (69.5)
Unknown	1 (1.7)	2 (3.4)

*IQR* interquartile range

The primary outcome of mean communication score 2 days after the information was given was lower in the telephone group than in the control group, effect size −0.74 (95% confidence interval (CI), −1.11 to −0.37). However, the participants of both groups had high scores of comprehension and satisfaction on an absolute scale (Table 2). When the scores of comprehension and satisfaction were analyzed separately, the mean score of comprehension in the telephone group was not significantly lower than in the control group, effect size −0.22 (95% CI, −0.58 to 0.14). In contrast, the mean score of satisfaction in the telephone group was significantly lower than in the control group (Table 2) effect size −0.87 (95% CI, −1.25 to −0.49). The reduction was based on a significant reduction in all the items of the satisfaction score. The effect sizes were slightly reduced when assessed 2.5 months later (Table 2).

**Table 2:** Results by allocation group

	Telephone group (*n* = 59)	Control group (*n* = 59)	Mean difference	*P* value
Primary outcome				
Scores, first questionnaire – mean (IQR)				
Total score (7–69)	61.40 (58 to 65)	64.42 (63 to 67)	−3.02	<0.001
Comprehension score (0–20)	16.90 (16 to 19)	17.31 (16 to 18)	−0.41	0.24
Satisfaction score (7–49)	44.51 (42 to 48)	47.12 (46 to 49)	−2.61	<0.001
Secondary outcomes				
Scores, second questionnaire – mean (IQR)				
Total score (7–69)	61.61 (58 to 66)	63.85 (61 to 68)	−2.24	0.024
Comprehension score (0–20)	16.83 (16 to 18)	17.31 (16 to 19)	−0.48	0.28
Satisfaction score (7–49)	44.78 (41 to 49)	46.54 (45 to 49)	−1.76	0.018
Length of interview – mean (IQR), min	8.9 (6 to 10.5)	12.1 (9 to 14)	−3.2	<0.001
			*χ* ^*2*^	*P* value
Accompanied by family member or friend at the information interview – number (%)	7 (11.9)	16 (27.1)	4.37	0.036
Sought more information after being informed – number (%)	29 (49.2)	23 (39.0)	1.24	0.27
Participating in The Danish Calmette Study – number (%)	50 (84.7)	54 (91.5)	1.42	0.49
Adherence to The Danish Calmette Study – number (%)				
Participating in 3-month follow-up	50 (84.7)	54 (91.5)	1.30	0.26
Participating in 13-month follow-up	48 (81.4)	54 (91.5)	2.60	0.11

*IQR* interquartile range, *min* minutes

Providing information in the telephone group took less time and fewer participants had a family member or friend present when the information was given compared to the control group (Table 2). We found no difference between the groups in the number of participants who sought more information about The Danish Calmette Study after the intervention of the present trial, the number of participants who ended up participating in The Danish Calmette Study, or in adherence to The Danish Calmette Study (Table 2)

## Discussion

In this randomized controlled trial we compared the effect of providing study information by telephone versus the standard face-to-face consultation, and found the combined score of comprehension and satisfaction to be lower in the telephone group. The difference was due to a lower satisfaction, while there was little difference in comprehension of the information given. Both groups scored high on satisfaction on an absolute scale, and less time was used on the telephone.

The strengths of the present trial lie in its randomized controlled design, and in utilizing the recruitment process of a large randomized clinical trial to evaluate the effect of giving study information over the telephone. Additionally, we used questionnaires corresponding to the validated QuIC for outcome measures and had a high adherence. However, the findings of the present trial may not be generalizable to all fields of research, as we studied a particular group; younger expectant mothers with a high educational level who were likely to be confident in communicating by telephone. Furthermore, our participants had expressed interest in hearing more about The Danish Calmette Study and, as such, were possibly more receptive to the information given. Another limitation of this trial is the timing of the second follow-up; ideally both the first and the second follow-up should be from the point of randomization. But, since the participants had the verbal information about The Danish Calmette Study repeated before they were randomized in it within 7 days after birth, this would have yielded big differences in time spans between the repetition of verbal information and the second follow-up in this trial. To ensure that the participants had approximately the same time span from verbal information to the second follow-up we chose 2.5 months after birth knowing that this might induce bias to the trial. Finally, this trial did not meet the recruitment target, since the trial had to stop when the recruitment for The Danish Calmette Study ended; however, the present trial remained powered to detect small differences between the randomization groups, also when examining comprehension and satisfaction separately.

A recent systematic review concluded that only enhanced Consent Forms and extended discussions were effective measures in improving participant comprehension [15]. One might assume that using less time to give the information in the telephone group could compromise the comprehension, though duration of discussions and comprehension are not necessarily linearly correlated. We found no difference in the comprehension between the two groups, or in the number of participants who sought more information after being informed. It should be noted that comprehension was generally high in both groups of our trial.

Similarly, the participants in both groups were very satisfied with the information process, although significantly less so in the telephone group. While satisfaction can be considered of less importance than comprehension from an ethical viewpoint, satisfaction may be important for study adherence. Furthermore, the satisfaction score included questions on disturbances and the opportunity for asking questions; both of which are important elements of obtaining informed consent. The reasons for the lesser satisfaction in the telephone group are probably multifaceted, but since the verbal information given was the same in both groups, nonverbal factors, such as body language or facial expressions, might have contributed as they are thought to be important determinants to understand or discern distress, confusion, disturbance, or attention.

We did not record the reasons for nonparticipation in our trial. Our impression is that the majority of those declining to participate did so because they preferred to be informed by telephone. Nonparticipants gave various reasons for this, e.g., it would take too much time to attend a consultation, logistical problems, and busy or inflexible schedules. This observation is in accordance with the findings of a recent Cochrane meta-analysis on improving the recruitment to randomized controlled trials. It found that telephone recruitment may increase potential participants’ willingness to participate relative to recruitment in person in a clinical setting [4, 16]. Other recent studies also found the use of the telephone in the informed consent process to be feasible [16–20]. Providing information on the telephone may be feasible, with the consideration that it may require additional efforts to obtain the participants’ satisfaction with the information process. Depending on how the telephone is used in the recruitment for clinical trials – as the only source of verbal information or as a supplement to face-to-face information – in could be used in both low- and high-risk trials. In high-risk trials, however, it is particularly important to consider the earlier-mentioned nonverbal factors.

Although we found that informing potential study participants on the telephone was feasible, no other study to our knowledge has examined the use of the telephone for informing study participants in a randomized design. Even though the use of the telephone for informing study participants may be an effective strategy, it should also be explored further whether it is ethically permissible especially for sensitive research subjects. Therefore, we encourage further research to confirm and extend our findings in other populations as well as efforts to further explore ethics in this important field that is relevant to all clinical researchers.

## Conclusion

In conclusion, the communication score was reduced in the telephone group which received verbal information by telephone. This was due to a reduction in satisfaction, while comprehension was similar to that of the control group which received the verbal information at a face-to-face consultation. This may be ethically acceptable, as both groups had very high scores on satisfaction on an absolute scale.

## Additional file

Additional file 1: Questionnaire 1 and Questionnaire 2, an English translation of the two questionnaires. (DOCX 21 kb)

## Abbreviations

QuIC: Quality of informed consent
