# Supplementary material for: Recruiting to Clinical Trials on the Telephone – a randomized controlled trial
Source: Trials. 2016 Nov 21;17:552. doi: 10.1186/s13063-016-1680-y (PMC5117558; doi:10.1186/s13063-016-1680-y)
Supplement: Additional file 1: — Questionnaire 1 and Questionnaire 2, an English translation of the two questionnaires. (DOCX 21 kb) [file 13063_2016_1680_MOESM1_ESM.docx]

# Additional file 1

| The columns show the content of Questionnaire 1, Questionnaire 2, the answers given, and comments on the minor quality improvements from the first to the second questionnaire as a result of further validation and response from participants. | | | |
| --- | --- | --- | --- |
| **Questionnaire 1** (Correct answer) | **Questionnaire 2** (Correct answer) | **Answer given to the participant** | **Comment** |
| **Prior knowledge of the Calmette study** (answered “Yes”, “No” or “Do not know”) | | | |
| *Have you heard of the Calmette study during antenatal classes?* | Not present in Q2. | None. |  |
| *Have you sought information about the Calmette study, beside what you were told at the information interview?* | Not present in Q2. | None. |  |
| *Were you accompanied by a family member or friend at the information interview?* | Not present in Q2. | None. |  |
| **Possible benefits of the Calmette vaccine** (answered “Yes”, “No” or “Do not know”) | | | |
| *It is certain that the Calmette vaccine protects against prevalent allergic diseases, asthma and atopic eczema.* (No) | Same as in Q1. | *No, it is not certain that the Calmette vaccine protects against prevalent allergic diseases, asthma and atopic eczema, but with the Calmette study this is what we are trying to investigate.* |  |
| *Does the research group think, that the Calmette vaccine can reduce the risk of childrens’ diseases among Danish children?* (Yes) | *The research group behind the Calmette study assumes that the Calmette vaccine is able to reduce the risk of children’s’ diseases like cold, pneumonia and diarrhoea among Danish children.* (Yes) | *Yes, this is the research group’s assumption on the basis of several studies from, among others, Guinea Bissau in Africa.* | Minor improvements of text clarity. |
| *The Calmette vaccine has a stimulating effect on children’s immune system.* (Yes) | Same as in Q1. | *Yes, some studies show that the Calmette vaccine stimulate some specific cells in the immune system.* |  |
| *The Calmette vaccine is a vaccine against measles.* (No) | Same as in Q1. | *No, the Calmette vaccine is a vaccine against tuberculosis.* |  |
| **Possible side effects of the Calmette vaccine** (answered “Yes”, “No” or “Do not know”) | | | |
| *The Calmette vaccine has been well tested and the side effects are well known.* (Yes) | *The Calmette vaccine is worldwide one of the most widely used vaccines and its side effects are well known.* (Yes) | *Yes, the Calmette vaccine has been used for about 100 years and is overall one of the world's most used vaccines through time.* | Minor improvements of text clarity. |
| *The Calmette vaccine’s most severe side effects are rare (<1 out of 1,000).* (Yes) | *The Calmette vaccine’s most common side effects are lymph node inflammation and severe allergic reactions.* (No) | *Q1: Yes.*  *Q2: No, lymph node inflammation and severe allergic reactions are, together with bone inflammation, classified as "uncommon side effects".* | This question and the following switched places since the most common adverse effect where mentioned prior to the most severe in our other information material. |
| *The Calmette vaccine’s most common side effects are lymph node inflammation and severe allergic reactions.* (No) | *The Calmette vaccine’s most severe side effects are rare (<1 out of 1,000).* (Yes) | *Q1: No, lymph node inflammation and severe allergic reactions are, together with bone inflammation, classified as "uncommon side effects". Q2: Yes.* |  |
| *The Calmette vaccine usually gives fever up to 5 days after vaccination.* (No) | Same as in Q1. | *No, fever is an uncommon side effect that <1 out of 100 children might get.* |  |
| *The Calmette vaccine causes redness, swelling and ulceration that begin 2-4 weeks after vaccination.* (Yes) | *After 2-4 weeks redness, swelling and ulceration begin to emerge in the area where the Calmette vaccination has been given.* (Yes) | *Yes, redness, swelling and ulceration begin to emerge after 2-4 weeks in the area where the Calmette vaccination has been given. The ulceration heals within a couple of months.* | Minor improvements of text clarity. |
| *The Calmette vaccine generally gives rise to a scar of around 2 cm in diameter.*  (No) | *The Calmette vaccine normally causes a scar with a diameter of about 2 cm.* (No) | *No, the Calmette vaccine results in a small, flat scar with a diameter of about 0.5 cm.* | Minor improvements of text clarity. |
| **Study objective and importance of the control group** (answered “Yes”, “No” or “Do not know”) | | | |
| *The Calmette Study is a medical research project about vaccines.* (Yes) | Same as in Q1. | *Yes.* |  |
| *In the Calmette study the participants themselves choose whether they will be in the vaccine group or the control group.* (No) | Same as in Q1. | *No, participants in the Calmette study are allocated to* either *the vaccination group* or *the control group. This is done by random distribution/draw.* |  |
| *All children participating in the Calmette study are vaccinated - one half receives a Calmette vaccine, while the other half receives a placebo vaccines (a dummy vaccine).* (No) | Same as in Q1. | *No, the children in the control group receive no dummy vaccine and thus they are not stung.* |  |
| *Only the vaccination group is followed by telephone interviews and clinical examinations.* (No) | Same as in Q1. | *No, there is the same follow-up in both groups.* |  |
| *There are clinical examinations at* either *3* or *13 months.* (No) | *There are clinical examinations,* either *when the baby is 3 months* or *when the child is 13 months.* (No) | *No, there are clinical examinations* both *at 3 months* and *at 13 months.* | Minor improvements of text clarity. |
| **Rights as a participant in medical research** (answered “Yes”, “No” or “Do not know”) | | | |
| *To approve a child's participation in the Calmette study, it is only necessary that one of the parents with custody sign the written consent form.* (No) | *To approve a child's participation in the Calmette study, all with custody of the child have to sign the written consent form.* (Yes) | *Q1: No, both holders of parental custody must sign the written consent form to participate in the Calmette study.*  *Q2: Yes, all with custody of the child have to sign the consent form.* | Since some of the participants in the Calmette study had only one parent with custody, this question has to be clarified to avoid discrimination. Thereby the correct answer changed from “No” to “Yes”. |
| *As a participant in the Calmette study, you can only withdraw from the trial if you request it by writing.* (No) | Same as in Q1. | *No, at any time and without cause, you can withdraw from the Calmette study.* |  |
| *If you say yes to participation in the Calmette study, you also allow the healthcare staff from the Calmette study access to your medical records.* (Yes) | *If you say yes to participation in the Calmette study, you also allow the healthcare staff from the Calmette study access to the mother’s and the child’s medical records*. (Yes) | *Yes, healthcare staff from the Calmette have access to the mother’s and the child’s medical records, for instance to indicate that you participate in the study.* | Minor improvements of text clarity. |
| *As participant in the Calmette study, information about your health and other private matters is subject to confidentiality.* (Yes) | Same as in Q1. | *Yes, all information about your health and other private matters is subject to confidentiality - also in the Calmette Study.* | Minor improvements of text clarity. |
| *As a participant in the Calmette study you are entitled to have a family member or friend present at the information interview.* (Yes) | Same as in Q1. | *Yes, as a research participant you are always entitled to have a family member or friend present at the information interview.* |  |
| **Participant satisfaction** (rated at a 7-point Likert scale) | | | |
| *How satisfied are you with the information interview in general?* | Same as in Q1. | None. |  |
| *How would you rate your option of asking questions during the interview?* | Same as in Q1. | None. |  |
| *How would you assess your options for reflection before you decided whether to participate in the Calmette study or not?* | Same as in Q1. | None. |  |
| *The information interview took place in an undisturbed environment without interruptions.* | Same as in Q1. | None. |  |
| *The information at the information interview was appropriate in scope and content.* | Same as in Q1. | None. |  |
| *After the information interview I felt well-informed about what it would mean to be part of the Calmette study.* | Same as in Q1. | None. |  |
| *Did you feel you were treated with dignity as a human being during the information interview?* | *Did you feel your were treated with respect during the information interview?* | None. | Minor improvements of text clarity. |
| Not present in Q1. | *Your satisfaction with being allocated to a given group of randomization (vaccination or control group).* | None. |  |
| Not present in Q1. | *Your acceptance of being allocated to a given group of randomization (vaccination or control group).* | None. |  |
| **Participation in the Calmette study** (answered “Yes”, “No” or “Do not know”) | | | |
| Not present in Q1. | *Are you participating in the Calmette study?* | None. |  |
| Not present in Q1. | *Has your child been Calmette vaccinated?* | None. |  |
| Not present in Q1. | *Has your child been Calmette vaccinated as part of the Calmette study?* | None. |  |
